# Effectiveness of selected issues related to used tyre management in Poland

**DOI:** 10.1007/s11356-022-18494-7

**Published:** 2022-01-10

**Authors:** Grzegorz Przydatek, Grzegorz Budzik, Małgorzata Janik

**Affiliations:** 1 Engineering Institute of Applied Sciences in Nowy Sącz, Zamenhofa 1a, Nowy Sącz, Poland; 2 Department of Machine Design, Rzeszów University of Technology, Powstańców Warszawy 8, Rzeszów, Poland

**Keywords:** Collection, Indicator, Recovery, Statistical analysis, Used tyres, Waste management

## Abstract

This paper discusses interactions between the generation, collection and recovery of used tyres while considering an indicator of their mass accumulation per area in Poland. Notably, this study aimed to assess selected issues related to used tyre management efficiency from 2008 to 2018 based on European Union and national regulations. Within 11 years, over 5 million Mg of used tyres was introduced into the domestic market—exceeding the amount required for 50 million registered vehicles. It was demonstrated that a significant tyre waste management process involved the recovery of 47% of all tyres, which was almost entirely correlated with the total volume of tyres. Only the growth trend for generated tyres was considered significant, and the rarely used indicator of the accumulation of used tyres per area exhibited an uneven accumulation of used tyres, with the highest amount being 48.06 Mg km^−2^ in a region with a small area but a significant volume of waste tyres. Therefore, the management of used tyres requires action in the country to optimally increase this form of waste collection while consolidating the development, gathering and processing infrastructure in the context of further minimising environmental pressure and increasing the efficiency of their use by considering the 4R principle.

**Supplementary Information:**

The online version contains supplementary material available at 10.1007/s11356-022-18494-7.

## Introduction

The development of the automotive industry worldwide favours the increasing global demand for tyres and the creation of a significant number of used tyres, which are considered waste due to their quantity and durability. Moreover, tyres do not degrade in the natural environment for up to 100 years (Gronowicz and Kubiak 2007). Generally, the disposal of used tyres and other polyisoprene-based products is problematic (Juma et al. 2006).

Hence, tyres have become a major source of waste on a global scale. Waste tyres arise from the operation of vehicles and the dismantling of end-of-life vehicles. The main components of tyres include rubber, fillers, soot, steel, sulphur, zinc oxide, process oil and vulcanisation accelerators, among others (Rofiqul Islam et al. 2010). Their related waste management includes collection, transport, treatment and disposal (including landfilling) (Przydatek and Krok 2018). However, landfilling is the most popular method of waste disposal, which wastes the energy and potential material from used tyres and poses risks in the form of environmental deterioration (Segre and Joekes 2000). Landfills pose a serious threat to the natural environment, firefighting and habitats for insects and rodents (El-Naqa 2005).

To apply the waste hierarchy, European Union (EU) member states have been adopting measures to encourage solutions that minimise environmental impacts (Pomberger et al. 2017). Therefore, on a global scale, all waste producers must be included in organised waste collection (Przydatek 2019). Such behaviour aims to help protect natural resources and prevent environmental degradation (Gharfalkar et al. 2015). One of the most popular models in Europe for improving used tyre management (and waste) considers optimisation and is based on the extended production responsibility (Sienkiewicz et al. 2012; Gaska et al. 2018).

Every year, a massive amount of waste is generated worldwide—especially in the form of used tyres. In China alone, it has been estimated that approximately 20 million Mg of tyres was generated in 2020 (Sun et al. 2016). Notably, the economy dealing with the alternative side of using waste is growing. Currently, the ideal solution for disposing of used tyres is recycling or incineration (Directive 2000/53/EC n.d.). In the rubber waste industry, tyres can also be used as a form of fuel, a component of bituminous mass and as materials in the roofing and paving industries (Silvestravieiete and Sleinotaite-Budriene 2002; Hernandez-Olivares et al. 2002). Another method of managing used tyres involves re-treading.

In 2013, the volume of used tyres in the EU reached 13.6 million Mg (Simić and Dabić-Ostojić 2017). Waste tyre management in the EU is regulated by the following legal acts: Council Directive 1999/31/EC (n.d.); Directive End-of-Life Vehicle 2000/53/EC (n.d.); Directive Waste Incineration 2000/76/EC (n.d.). According to these acts, vehicle parts (including tyres) must be reused, recycled or recovered, while the disposal of whole and shredded car tyres is prohibited. However, EU member states have the option of choosing a waste tyre management system that considers the relevant fees and free market (Sienkiewicz et al. 2012).

The product life cycle—or, more precisely, the life cycle of the tyre—consists of intangible and tangible stages. The first stage includes design and construction. The second stage consists of three phases: manufacturing, use and disposal of the used tyre. In all phases of the material cycle, tyres harm the natural environment and human health while exhausting non-renewable resources. In contrast, Clauzade et al. (2010) and Torretta et al. (2015) found that all car tyre recycling/recovery methods provide environmental benefits.

Used tyres are remanufactured, recycled or co-incinerated in cement plants as an alternative fuel. Used tyre recycling is an extremely difficult process due to the diversity of raw materials from which they are produced (Sienkiewicz et al. 2012). To reduce their impact on the environment, tyres should be disposed of through incineration, which is the fastest and easiest tyre disposal procedure (Machin et al. 2017).

The main reasons for neutralising waste include economic development and the improvement of living standards in society. As a result, waste and alternative technical solutions are becoming increasingly modern in their development.

According to the hierarchy of waste management, the prevention of waste is required. When this is impossible, it is necessary to ensure waste recovery and recycling while preventing landfilling (COM 2005). The landfilling of used tyres is prohibited, except for bicycle tyres and tyres with an outer diameter greater than 1,400 mm (Act on Waste 2012). A valuable element in the management of used tyres is energy recovery (Huang et al. 2012).

Unfortunately, since many tyres are damaged or destroyed, a method of recycling them must be found to give them a new shape or function. Too much of this type of waste causes excessive accumulation, which makes recovery less likely. Rubber, steel and textiles that are suitable for reuse or energy generation are eligible for recovery. The recycling of used tyres through the course of recovery is aligned with environmental protection.

The present study aimed to assess the efficiency of selected issues in waste tyre management in Poland from 2008 to 2018 by considering waste tyre generation, collection, recovery and mass accumulation per area.

## Description of the study country

Poland is a country located in Central Europe. The country ranks 69th in terms of its area (312,696 km^2^), 36th in terms of global population and ninth in terms of population within Europe. In administrative terms, Poland is divided into three levels: voivodeship, district and commune. The largest area (35.579 km^2^) is occupied by the Mazovian Voivodeship in the central part of the country and has the greatest number of inhabitants, while the smallest area (9.412 km^2^) is occupied by the Opolskie Voivodeship in the south-western part (Przydatek 2020). The lowest average population densities are present in the Podlaskie and Warmian-Masurian voivodeships.

## Used tyre management in the country

**Fig. 1:**
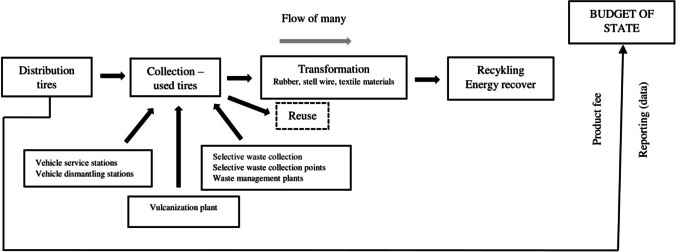
Diagram describing used tyre management in Poland

In Poland, the used tyre collection system is primarily implemented by vehicle service stations and end-of-life vehicle dismantling stations (NWMP 2016). Used tyres can also be delivered by individuals to a selected vulcanisation plant. Depending on the location, used tyres can be returned for free or for a fee. Used tyres can also be collected during selective waste collection as bulky waste (Skrzyniarz 2020). In contrast, certain taxes are levied in some countries, such as Denmark, Slovakia and Croatia (Kaur et al. 2021). A significant supplement to the collection of used tyres includes selective waste collection points and waste management plants (Fig. 1). Despite the Mazovian Voivodeship registering the most vehicles, the largest collection point for used tyres is in the Greater Poland Voivodeship (CRVD-Central Register of Vehicles and Drivers 2018,).

Many tyre collection points exist throughout the country due to the ban on landfilling (waste code 16 01 03—used tyres) (Regulation 2014). A total of 60 plants deal with the management of used tyres; however, few of them deal with comprehensive disposal or the recycling of materials. In the Opolskie Voivodeship, an installation enables the co-combustion of used tyres. According to Wasilewski and Stelmach (2009), this process is favoured by the significant calorific value of tyres (31.4 MJ kg^−1^). One of the factors aimed at reducing the amount of waste generated in both Poland and other EU countries is the use of the waste hierarchy (including recycling), which reduces the effect of waste on the environment, the consumption of natural resources and the costs (Eriksson et al. 2005).

## Methods and materials

Data acquisition was based on a questionnaire that was addressed to 16 individual Provincial Marshal Offices in Poland and also included owner observations. Based on annual data for 2008 to 2018 that included the total sum and quantities of generated, collected and recovered tyres, an analysis was performed that involved determining the mass accumulation indicator of tyres by area (division by country and voivodeship in Mg km^−2^). The waste accumulation indicator by area was used in studies by Przydatek and Ciągło (2020) and Xiao et al. (2012). Generated waste tyres are primarily produced in vulcanisation plants, service points and dismantling stations for end-of-life vehicles (NWMP 2016).

According to Miliute-Plepiene and Plepys (2015), the number of studies considering waste accumulation indicators is increasing, which may result from the need to identify factors causing an increase in the amount of generated waste. Additionally, the number of registered vehicles in Poland was determined based on figures published by Statistics Poland 2008-2018). This number included motor vehicles, buses, lorries, special cars, tractor units, agricultural tractors, motorcycles and mopeds.

A statistical analysis was also performed that involved calculating the maximum, minimum and arithmetic average. To determine the correlation meeting the condition of a normal distribution for data covering the total number of tyres—including those generated, collected and recovered—the Pearson linear correlation coefficient method was used. When the condition of a normal distribution was not met, Spearman’s rank method was applied. The Spearman correlation coefficient *R* is a non-parametric equivalent of Pearson’s coefficient. As per a parametric correlation, the Spearman correlation coefficient *R* measures the strength of the correlation between variables. Non-parametric tests were used due to the lack of normality of the distribution of most analysed indicators following the results of the Shapiro–Wilk test (*p* < 0.05) (Przydatek and Kanownik 2019).

The non-parametric Mann–Kendall statistical test was chosen to test a series of numbers to identify an upward or downward trend that is not necessarily linear. Statistica 13 (StatSoft Poland, StatSoft, Inc., USA) was used for statistical analysis.

## Used tyre quantity analysis

The total volume of used tyres collected in Poland from 2008 to 2018, divided by the 16 individual voivodeships, is listed in Table 1. The smallest mass of collected tyres was recorded in the north-eastern part of the country (Podlaskie Voivodeship), while the highest was recorded in the central-western region of Poland (Greater Poland Voivodeship; area: 35.66 Mg km^−2^). These values were 42,067 Mg and 1,063,657 Mg. In contrast, the highest amount of used tyre accumulation (48.06 Mg km^−2^) occurred in the southern part of central Poland (Świętokrzyskie Voivodeship; ranked 15th in Poland in terms of size; 562,848 Mg), whereas the lowest was 1.94 Mg km^−2^ in the north-eastern part of the country (Warmian-Masurian Voivodeship; ranked fourth in Poland in terms of size; 46,983 Mg) (Table 1). The total amount of tyres during the 11 analysed years ranged from 708.24 to 135,570 Mg, with an average of 32,741 Mg (Table 2).

**Table 1:** Total amount of used tyres divided into administration regions of Poland

Voivodeship	Amount of tyres	Tyre’s accumulation
	Mg	Mg km^−2^
Lower Silesia	116,813	5.86
Kuyavian-Pomeranian	175,155	9.75
Lublin	427,502	17.02
Lubusz	595,164	42.55
Łódz	327,739	17.99
Lesser Poland	348,935	22.98
Mazovian	234,703	6.60
Opole	446,564	47.45
Podkarpackie	548,455	30.73
Podlaskie	42,067	2.08
Pomeranian	109,527	5.98
Silesian	209,118	16.96
Świętokrzyskie	562,848	48.06
Warmian-Masurian	46,983	1.94
Greater Poland	1,063,657	35.66
West Pomeranian	159,434	6.96
Min	42,067	1.94
Max	1,063,657	48.06
Average	338,416	19.91

**Table 2:** Average amount of generated, collected and recovered used tyres in Poland

Indicator	Average	Min	Max
Mg			
Amount of tyres*	32,741	708.24	135,570
Collected*	14,295	73.16	80,197
Recovered*	16,060	0.50	58,401
Generated*	3,950	467.02	38,134
pcs			
Vehicles**	52,471,172	43,389,232	62,570,032

^*^On the base data from voivodeships

^**^On the base data from Statistics Poland

**Fig. 2:**
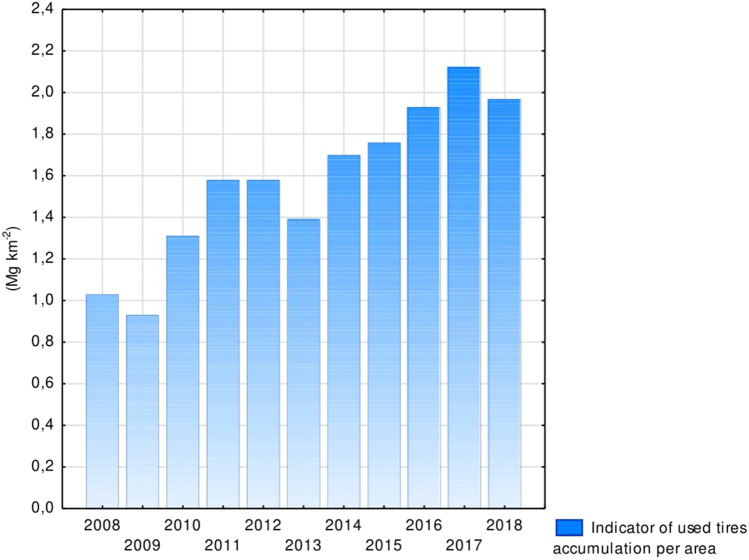
Indicator of used tyre accumulation per area

**Fig. 3:**
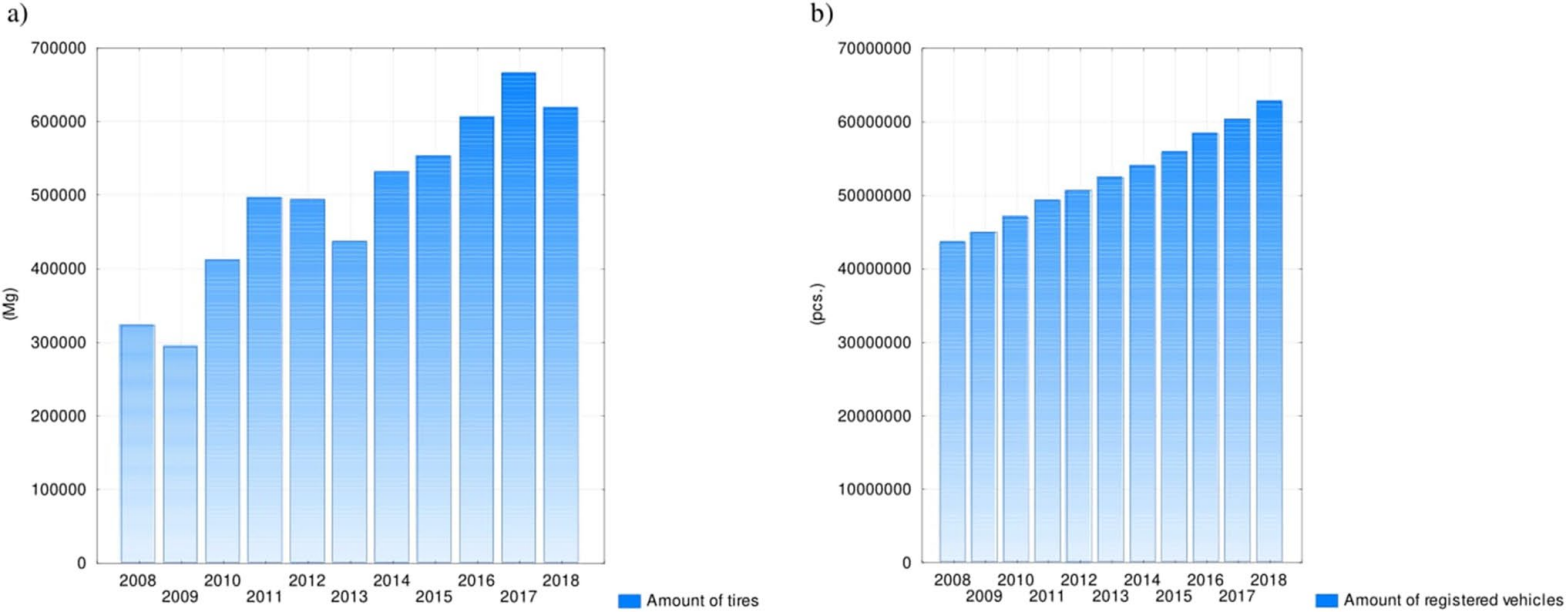
Amount of collected and used tyres

In the analysed years, the accumulation indicator of collected tyres per unit area in the country ranged between 0.93 and 2.12 Mg km^−2^ (Fig. 2). During this period, the lowest total amount of waste tyres was 292,277, while the highest was 663,935 (Fig. 3a). Moreover, the number of vehicles registered ranged from 43,389,232 to 62,570,032, which increased by as much as 19,180,800 (Fig. 3b). The highest number of vehicles (284,065,550) comprised motor vehicles and tractors (49.22%), while the lowest number (1,152,220) was for buses (0.20%). The most extreme values for the accumulation rate and the number of used tyres were recorded in 2009 and 2017, respectively (Statistics Poland 2008-2018).

**Fig. 4:**
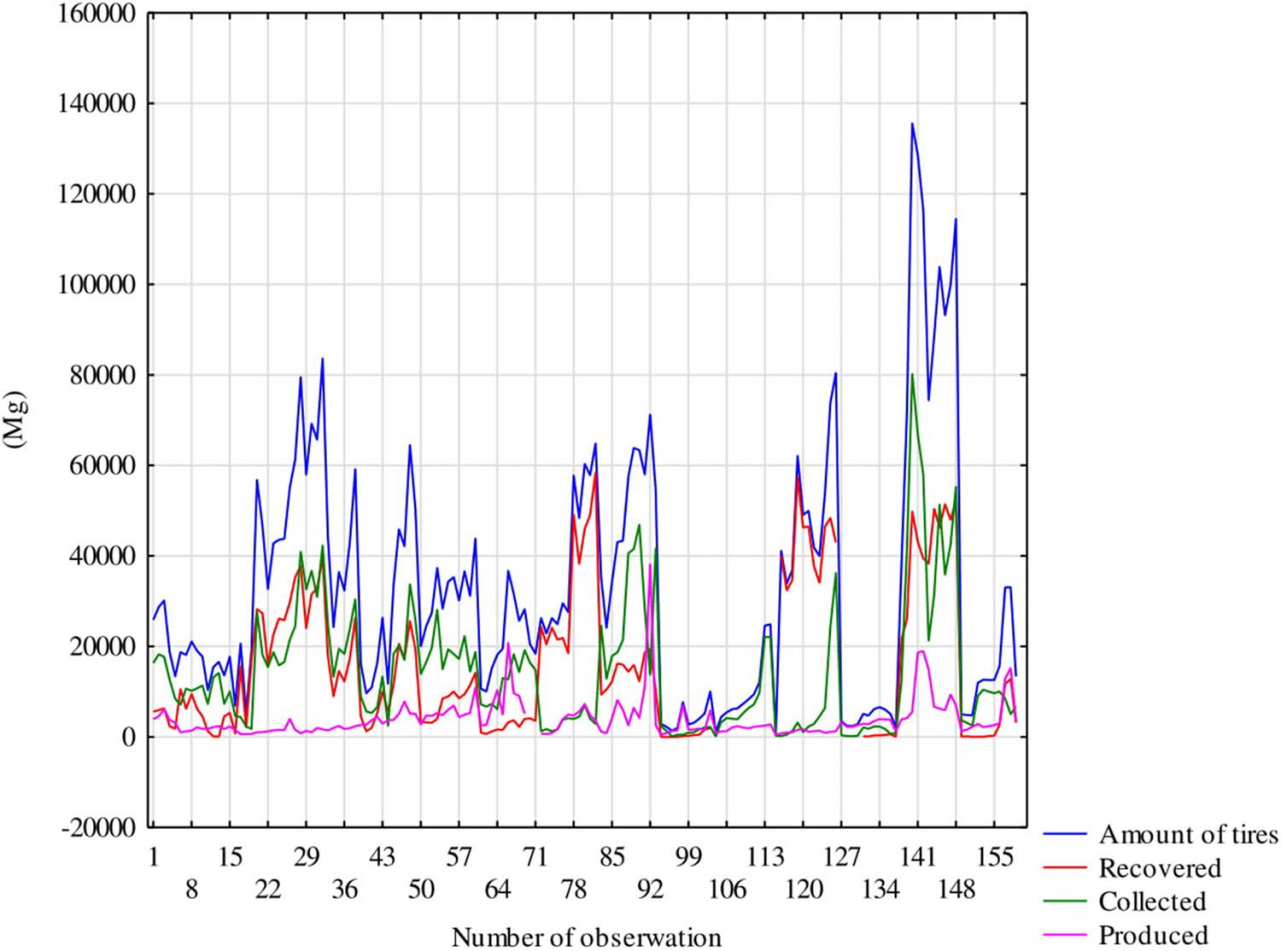
Linear variation in the value

**Fig. 5:**
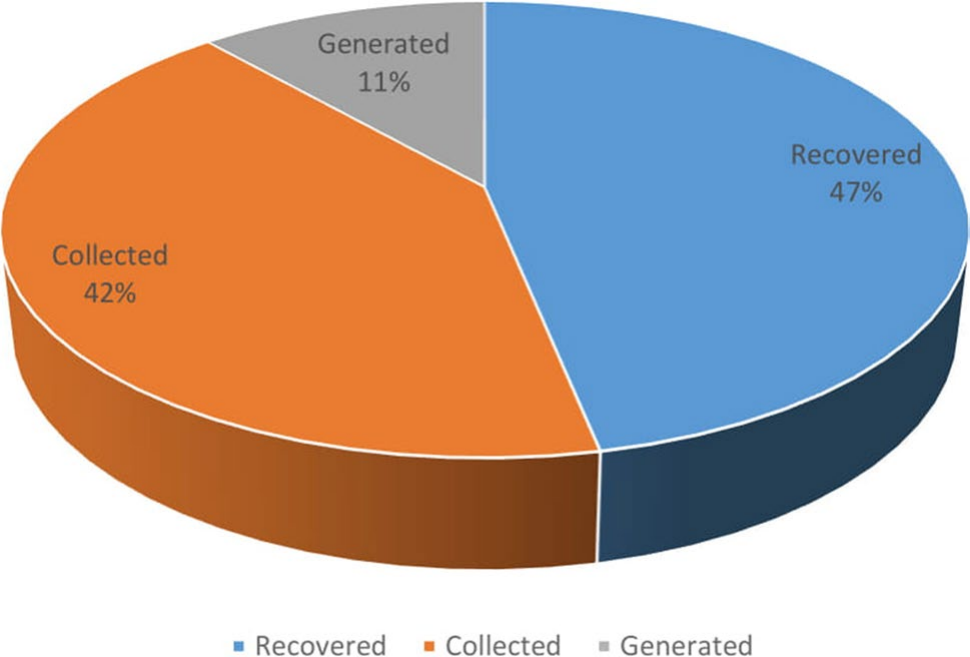
Share of individual processes used tyre management

The variability of the indicated data is presented in Fig. 4, with recovered tyres representing 47% and generated used tyres having the lowest share (11%; Fig. 5). However, the maximum values differ significantly in this respect. The highest value of 80,197 Mg was recorded for collected tyres (excluding the total amount of tyres).

The average values of collected and recovered tyres were 14,295 and 16,060 Mg, respectively, with a total amount of 32,741 Mg. The lowest average amount was recorded for generated tyres (3,950 Mg) (Table 2).

The highest correlation value (0.90) was calculated between the total tyre and recovered tyre amounts. Moreover, the correlation value of 0.73 calculated between the total number of tyres and collected tyres was high. Furthermore, there was a moderate correlation (0.43) between the number of recovered and collected tyres. The remaining relationships (i.e. between the number of collected and generated tyres, total quantity of collected and generated tyres, and the number of recovered tyres) did not exceed 0.4 (Table 3).

**Table 3:** Correlation between variability of amount generated, collected and recovered used tyres

Tyres	Total amount	Recovered	Collected	Generated
Total amount		*0.90*	*0.73*	*0.30*
Recovered	*0.90*		0.43	0.04
Collected	*0.73*	*0.43*		*0.38*
Generated	*0.30*	0.04	*0.38*	

^a^Italic value of statistics means that the relationship is statistically significant at *p* < 0.05

However, the trend observed in this study only confirmed a statistically significant increase in the number of generated tyres and a decrease in the number of collected tyres (Table 4).

**Table 4:** Time trends of generated and collected waste tyres

Variable	Trend	Probability (*p*)
Collected tyres	↓	*0.01*
Generated tyres	↑	*0.02*

## Discussion

In Poland, the total amount of used tyres over 11 years exceeded 5 million Mg, with an average of 32,741 Mg. According to Karaağaç et al. (2017), the annual amount of waste tyres in Turkey was estimated to be greater than 250,000 Mg. Similarly, in Greece, the amount of collected waste tyres exceeded 50,000 Mg (Panagiotidou and Tagaras 2005).

During their life cycle, the ecotoxic effect of tyres on the environment results from their zinc, nickel, copper, lead, chromium and copper content (Piotrowska et al. 2019). Formela et al. (2016) demonstrated that the environmental impact of used tyres depends on their structure (i.e. traditional materials or natural rubber) (Uruburu et al. 2012).

One of the most important aspects of waste management is the prevention of waste. However, this is a complex problem in the case of used tyres because the increase in the number of used tyres depends on the number of vehicles in use. The average number of vehicles in the analysed period was over 1.5 times higher than the number of tyres collected. As the number of vehicles increases, so does the number of tyres and their waste (Yadav and Tiwari 2017). Despite a successive increase in the number of vehicles, the largest amount of used tyres was recorded in 2017. This result was observed in the central-western part of Poland, where the largest number of tyre collection points is located. De Figueiredo and Mayerle (2008) noted that the level of used tyre recycling depends on optimising the number and location of collection points. However, despite its favourable results, the reuse of rubber as part of recycling in EU countries becomes problematic due to the declining demand for granules as a result of the economic crisis (Torretta et al. 2015). In contrast, Karaağaç et al. (2017) indicated that the demand for polymeric materials has been increasing in Turkey over the recent years.

**Fig. 6:**
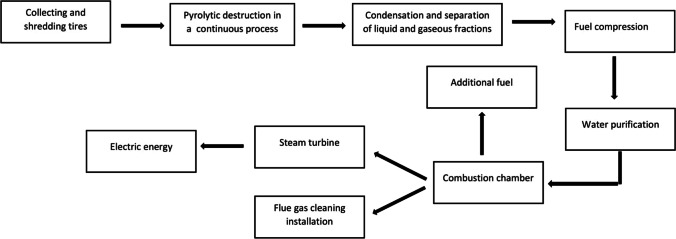
Process of recovery energy from used tyres

According to Skarbek and Michalski (2012), the effective management of used tyres involves recovery and recycling. As part of the recycling process, waste tyres are used to produce bituminous mass, which allows lower raw material costs (Hsisheng et al. 1995). Ahn and Cheng (2014) demonstrated that most tyres are not adequately managed by landfilling, resulting in ‘wild’ landfills. Moreover, Isse and Salem (2013) classified combustion as one of the most popular methods of tyre management, the side effect of which is the emission of pollutants into the atmosphere. According to Machin et al. (2017), the uncontrolled combustion of used tyres generates 6% of the pollution caused by releasing toxic fumes into the atmosphere and also leads to water pollution through runoff. In Italy, approximately two-thirds of energy is produced in this manner (Torretta et al. 2015). The recovery of this energy is most often performed via the direct burning of tyres (fragmented or whole) or in the pyrolysis process (Fig. 6), which is one of the effective processes of thermal conversion of waste with the calorific value of the pyrolytic liquid within the range of 41 to 44 MJ kg^−1^ (Williams 2013). Godlewska (2017) noted that waste tyre recovery most often implies energy recovery.

In turn, the recovery of used tyres in Poland was significant at approximately 47%. This result indicates that risk to the environment is minimised when following the 4R principle (Mmereki et al. 2016). In Ecuador, Cecchin et al. (2019) recorded a lower level of used tyre recovery (20%). Notably, the reuse of tyres through re-treading is important. In this respect, Poland ranks seventh in the EU (ETRMA European Tyre Rubber Manufactures Assocciation 2014). However, Poland’s share of generated tyres was low (11%). In Lebanon, the highest production of waste tyres was observed in their third year of use (Mrad and El-Samra 2020).

The collection of tyres is also important. In the present study, the maximum value of collected tyres exceeded the recovery by over 20,000 Mg. However, the recovery of used tyres as waste in the form of recycling is considered significant by some researchers (Djadouni et al. 201) because it can reduce the quantity of assembled waste (Przydatek 2016).

An almost complete correlation relationship was observed between the total amount of tyres and their recovery. Despite this, this parameter indicated a significantly increasing trend, whereas tyre collection exhibited a significant decline. Additionally, Rafique (2012) observed an increase in the number of generated waste tyres in Poland. Moreover, Uruburu et al. (2012) presented a positive trend in Eastern Europe, where the secondary use of rubber is increased for hardening roads with modified asphalt. Pastor et al. (2014) drew attention to the possibility of using waste tyres as noise barriers, artificial barriers and bales after their disintegration.

Furthermore, Przydatek and Ciągło (2020) considered the indicator of waste accumulation in an area to be important since this indicator may help to assess tyre management in terms of selecting the locations for treatment plants and the number of tyre waste collection points. The tyre accumulation per country area in the analysed multiannual period reached a maximum value of 2.12 Mg km^−2^. The accumulation result of 48.06 Mg km^−2^ was higher, with the amount exceeding 500,000 Mg in one of the smallest voivodeships in terms of area and the number of waste collection points. For comparison, the largest voivodeship in Poland had the greatest number of registered vehicles. Based on current trends in such fields, noticeable variability in the accumulation of used tyres should enable the selection of solutions conducive to rational management while minimising the negative effects of tyres on the environment. According to Kaur et al. (2021), energy can be recovered from used tyres, which can help reduce their negative environmental effects.

## Conclusions

The analysis of test results for the total amount of used tyres—including those generated, collected and recovered in Poland—resulted in the following conclusions:

- Under EU requirements and the hierarchy of waste management in Poland, tyres were reused, recovered and recycled.
- The significant average amount of used tyres (32,741 Mg) confirms that this form of waste poses a potential problem in the area of environmental protection.
- The average number of vehicles in relation to the number of used tyres (over 1.5 times higher) shows a trend of noticeable growth in the number of generated tyres.
- The greatest volume of used tyres was recorded in 2017, despite the highest number of registered vehicles being recorded in 2018 and the significant quantity of used tyres in the central-western part of the country, which had the largest number of tyre collection points.
- The tyre accumulation indicator per country area should be considered very helpful because it demonstrated significant differentiation between the result achieved on the national scale (2.12 Mg km^−2^) and that achieved in one of the smallest voivodeships (48.06 Mg km^−2^). These results suggest a need to increase the number of points in other parts of the country that can accept used tyres (as part of a bulky waste collection).
- The recovery rate of used tyres (47%) showed a moderate correlation with waste tyre collection, whereas the correlation with the total amount of tyres was nearly perfect. This confirms the need to develop the selective collection of used tyres due to the decreasing trend of tyre collection.
- The desirable and simultaneously effective direction for the utilisation of used tyres should be their efficient thermal conversion with energy recovery.

## Supplementary Information

Below is the link to the electronic supplementary material.

Supplementary file1 (PDF 105 KB)

## Data Availability

The datasets used and/or analysed during the current study are available from the corresponding author on reasonable request All data generated or analysed during this study are included in this published article (and its supplementary information files).
