# Supplementary material for: Effectiveness of selected issues related to used tyre management in Poland
Source: Environ Sci Pollut Res Int. 2022 Jan 10;29(21):31467–75. doi: 10.1007/s11356-022-18494-7 (PMC9054868; doi:10.1007/s11356-022-18494-7)
Supplement: Supplementary file 1 — Supplementary file1 (PDF 105 KB) [file 11356_2022_18494_MOESM1_ESM.pdf]

EDITORIAL CERTIFICATE

Authors:

Grzegorz Przydatek
Grzegorz Budzik
Małgorzata Janik

Document title:

Effectiveness of selected issues of used tyre management in Poland

Date Issued:

22 Dec 2021

Cambridge Proofreading LLC

This document certifies that the above manuscript was proofread and edited by Cambridge Proofreading LLC.

This document certifies that the above manuscript was proofread and edited by Cambridge Proofreading Worldwide LLC. The document was edited for proper English language, grammar, punctuation, spelling, and overall style by one or more of our academic editors. The editor endeavoured to ensure that the author's intended meaning was not altered during the review. All amendments were tracked with the Microsoft Word 'Track Changes' feature. Therefore, the authors had the option to reject or accept each change individually.

Kind regards,
Cambridge Proofreading

CAMBRIDGE PROOFREADING LTD
EDITORIAL CERTIFICATE

Cambridge Proofreading Worldwide LLC is a registered company headquartered in Chicago, Illinois, USA with a global presence. All of our editors are native speakers from USA and the UK. Our Certificate of Good Standing can be found in the Illnois state business database by searching our name here.
